# Serum neurofilament light chain and inflammatory cytokines as biomarkers for early detection of mild cognitive impairment

**DOI:** 10.1038/s41598-024-59530-5

**Published:** 2024-04-20

**Authors:** Xinyang Jing, Lan Wang, Mei Song, Hao Geng, Wei Li, Yaxin Huo, Anqi Huang, Xueyi Wang, Cuixia An

**Affiliations:** 1 Mental Health Center, The First Hospital of Hebei Medical University, Hebei Clinical Research Center for Mental Disorders and Institute of Mental Health, Hebei Technical Innovation Center for Mental Health Assessment and Intervention, 89 Donggang Road, Shijiazhuang, 050031 China; 2 Hebei Key Laboratory of Forensic Medicine, Shijiazhuang, 050017 Hebei Province China

**Keywords:** Neuroflament light, Inflammatory cytokines, Mild cognitive impairment, Alzheimer’s disease, Biomarkers, Biomarkers, Medical research

## Abstract

To investigate the association between serum neurofilament light chain (NfL) levels, inflammatory cytokines, and cognitive function to assess their utility in the early detection of mild cognitive impairment (MCI). We conducted a cross-sectional study involving 157 community-dwelling individuals aged 55 years and above, categorized into healthy controls, MCI, and probable Alzheimer's disease (AD). Serum levels of NfL, inflammatory cytokines, and AD pathology markers were measured using enzyme-linked immunosorbent assay (ELISA). Correlations between these biomarkers and cognitive function were analyzed, and the diagnostic performance of the cognitive assessment scales and serum biomarker concentrations was evaluated using receiver operating characteristic (ROC) curve analysis. Serum NfL levels were significantly elevated in MCI and probable AD groups compared to healthy controls. Positive correlations were found between serum NfL and inflammatory cytokines IL-1β, IL-6, and Aβ40. Combining serum NfL with p-tau217 and the Boston Naming Test significantly enhanced the predictive accuracy for MCI. However, combining serum NfL with inflammatory markers did not improve MCI prediction accuracy. Elevated serum NfL is associated with cognitive impairment and inflammatory markers, suggesting its potential as a peripheral serum biomarker for MCI detection. The combination of serum NfL with p-tau217 and cognitive tests could offer a more accurate prediction of MCI, providing new insights for AD treatment strategies.

## Introduction

Alzheimer's disease (AD) is a neurodegenerative disorder hallmarked by the accumulation of amyloid-beta (Aβ) and tau proteins, brain tissue atrophy, and progressive cognitive decline^1^.The global prevalence of AD is escalating in tandem with the aging population. The International Alzheimer's Disease Association reported approximately 46 million individuals with AD worldwide in 2018, with projections suggesting a rise to 131.5 million by 2050^2^. In China, recent epidemiological data indicates a dementia prevalence of 6.04%, with AD accounting for 3.94% among those over 60, translating to nearly 9.83 million AD patients and an increasing socio-economic impact^3^.

The challenge of timely and accurate dementia diagnosis is formidable, with an estimated three-quarters of dementia cases remaining undiagnosed globally, a figure that could be as high as 90% in low- and middle-income countries. Current diagnostic markers, such as cerebrospinal fluid (CSF) Aβ and tau proteins, along with neuroimaging techniques like MRI, tau-PET, β-amyloid-PET, and F-FDG-PET, facilitate early AD detection but are limited by invasiveness, cost, and accessibility^4^. Consequently, there is an urgent need for less invasive and more cost-effective biomarkers.

Neurofilaments, specifically the light chain (NfL), have garnered attention as potential peripheral biomarkers for neuronal damage. Neurofilaments are neuron-specific, highly phosphorylated proteins that form a key component of the axonal cytoskeleton, with NfL being a critical subunit involved in axonal growth and stability^5^. Under physiological conditions, axonal cells release minimal amounts of NfL into the CSF, which can then enter the bloodstream, albeit at lower concentrations. However, pathological conditions trigger a marked increase in NfL, making it a sensitive marker for axonal injury^6^. Elevated CSF NfL levels have been associated with cognitive impairment, with studies demonstrating its rise in early AD and its correlation with cognitive decline and brain structural changes^7^. A study has found that the content of NfL is correlated with age, and NfL concentrations increased at a rate of 3.1% per year of age. Elevated CSF NfL has also been observed in other dementias, correlating with lower Mini-Mental State Examination (MMSE) scores and reduced survival times^8^. Furthermore, higher NfL levels have been linked to cognitive decline in mild cognitive impairment (MCI) and AD patients, irrespective of vascular burden^9^. Furthermore, a longitudinal study showed that the concentration of light filament chains at baseline predicted future cognitive performance in patients with small cerebral vessels^10^, raising the possibility that light filament chains could be used to predict future cognitive function in dementia.

Given the invasive nature of CSF collection, the focus has shifted towards blood-based NfL measurements. Because the use of Single Molecule Array (Simoa) technology analysis platform limits the feasibility of NfL as a global biomarker, we choose the simpler and more operable ELISA in terms of measurement method. Significant correlations between plasma NfL and CSF biomarkers have been reported, with increased plasma NfL levels observed in MCI and AD, suggesting its potential as a non-invasive biomarker for cognitive impairment^1,11^. The relationship between NfL and cognitive function in the older adults is further supported by its role as an axonal damage marker, which is closely related to microglial activation in the central immune system^12^. Inflammation is increasingly recognized as a contributor to neurodegeneration, with systemic elevations of pro-inflammatory cytokines like TNF-α implicated in cognitive decline in AD^13,14^. The dysregulation of inflammatory pathways in MCI may also serve as a predictor for the rate of cognitive deterioration^15^. While there is evidence of a strong correlation between plasma NfL and both peripheral and cortical inflammation during AD progression, and suggestions of AD as a chronic autoimmune condition with heightened pro-inflammatory factors^16,17^, research into the relationship between blood NfL and inflammatory markers remains limited. More than 40 years ago, the Chinese government introduced policies to carry out educational reforms, which not only focused on primary and secondary education, but also placed greater emphasis on university education, providing many people with opportunities to receive higher education. This had led to the diversity of cultural levels among elderly residents in the community. Therefore, based on this population, this study explores the role of serum NfL and inflammatory cytokines in early detection of MCI, and clarify the relationship between serum NfL and inflammatory markers.

## Materials and methods

### Study design and participants

This cross-sectional study was conducted from April 2021 to February 2022 in Hebei Province, China. Participants were recruited from the Yuhua District of Shijiazhuang City and Renze District of Xingtai City, resulting in a cohort of 157 older adults aged 55 years and older, comprising 74 males and 83 females. All participants were asked if they were willing to participate in this study and each person signed an informed consent form. The study was approved by the Ethics Committee of the First Hospital of Hebei Medical University (ethical approval number: 20190416). We confirm that all research were performed in accordance with relevant guidelines and regulations.

### Inclusion and exclusion criteria

Inclusion criteria for the study were as follows: (1) age 55 years or older; (2) willingness and ability to participate in and complete a comprehensive questionnaire survey and cognitive function evaluation; (3) consent to provide blood samples for analysis. Exclusion criteria were established to omit individuals with potential alternative causes of cognitive decline, including those with a clear history of stroke, brain tumor, Parkinson's disease, epilepsy, severe head trauma, and significant mental disorders. Additionally, individuals with severe physical illnesses or substantial visual or hearing impairments that could interfere with cognitive function testing were excluded.

### Diagnostic criteria

Participants were classified into two groups: Healthy older adults with normal cognition (healthy control, HC) and those with cognitive impairment, and the cognitive impairment group was further divided into the MCI group and the probable AD group.

HC Group Diagnostic Criteria:

1. Objective evidence of normal cognitive function, stratified by educational level with respective cut-off scores on the MMSE: ≥ 19 points for illiterate, ≥ 22 points for elementary school, and ≥ 24 points for junior high school and above^18^. Additionally, Montreal Cognitive Assessment (MoCA) scores of ≥ 13 points, ≥ 19 points, and ≥ 24 points were required for the respective educational levels^19^.
2. Normal daily living abilities, as indicated by a score of ≤ 26 points on the Activity of Daily Living Scale (ADL)^20^.

MCI Diagnostic Criteria:

As per the consensus established by the Chinese Expert Group on Cognitive Impairment Prevention and Treatment in 2006^21^ , MCI diagnosis included:

1. Self-reported or family-observed significant memory impairment.
2. Preservation of other cognitive functions or only mild impairment, with MMSE scores of ≥ 19 for illiterate, ≥ 22 for elementary school, and ≥ 24 for junior high school or above. Corresponding MoCA scores should be < 13, < 19, and < 24, respectively.
3. Maintenance of basic daily living activities, with an ADL score ≤ 26.
4. Exclusion of dementia, ensuring that the criteria for dementia diagnosis are not met.
5. Exclusion of other systemic diseases that could affect cognitive function.

Probable AD Diagnostic Criteria^22^:

1. Documented history of cognitive decline, as evidenced by reduced scores on neurocognitive tests, specifically MMSE scores of < 19 for illiterate, < 22 for elementary school, and < 24 for junior high school or higher education.
2. Slight impairment in daily living activities, with an ADL score > 26.

### Data collection procedures

Trained survey personnel conducted the data collection process, which included administering a custom-designed general information survey form to collect demographic data such as age, gender, education level, smoking and alcohol consumption habits, and medical history.

### Neuropsychological assessments

Cognitive function was assessed using the MMSE^18^ and the MoCA^19^. The MMSE is a widely utilized tool that evaluates nine cognitive domains, including temporal and spatial orientation, immediate memory, calculation, delayed recall, naming, repetition, comprehension, and visuospatial abilities, with a maximum score of 30 points. The scoring is adjusted for cultural differences.

The MoCA is known for its higher sensitivity in detecting mild cognitive deficits compared to the MMSE. It evaluates several cognitive domains: visuospatial and executive functions, naming, memory, attention, language, abstraction, delayed recall, and orientation.

Additionally, the Boston Naming Test (BNT)^23^ was used to assess language production, specifically the ability to name pictorial representations, and the Digit Span Test (DST)^24^ measured immediate memory, attention, and processing speed.

### Blood sample collection and processing

Blood samples were collected from participants following an overnight fast. Standardized venipuncture procedures were performed in the morning by trained nurses, who collected 5 mL of venous blood from each participant in EDTA vacuum collection tubes (Inspeck, ST750EK, Sekisui, Osaka, Japan) at 8:00 a.m. in the morning. The whole blood samples were allowed to clot at room temperature for 2 h before centrifugation at 3000 rpm for 10 min^25^. The resulting serum was aliquoted using a serum separator tube and stored at − 80 °C until analysis, to avoid repeated freeze–thaw cycles.

Serum concentrations of NfL, Interleukin-1β (IL-1β), Interleukin-6 (IL-6), Tumor Necrosis Factor-α (TNFα), Amyloid-β40 (Aβ40), Amyloid-β42 (Aβ42), and phosphorylated tau protein 217 (p-tau217) were measured using enzyme-linked immunosorbent assay (ELISA) kits (Product IDs: CSB-E16094h, EK101B-24, EK106/2-24, EK182-24, CSB-E08299h, CSB-E10684h, ZC-55233, respectively). All kits are from ZCIBIO Technology Co.,Ltd, Business license code 91310120MA1HM97U98.

The ELISA procedure was conducted as follows: Reagents were equilibrated to room temperature (18–25 °C) for at least 30 min. Following the manufacturer's instructions, reagents were prepared and added to the ELISA plate wells, with 100 μl of standard or test sample per well. After mixing and sealing, the plates were incubated at 37 °C for 2 h. Post-incubation, the liquid was discarded, and the wells were washed three times with a 2-min soak of 200 μl per well. Subsequently, 100 μl of biotin-labeled antibody working solution was added to each well and incubated for another hour at 37 °C. Following this, the wells were washed five times, and 100 μl of horseradish peroxidase-labeled avidin working solution was added to each well. After a final hour of incubation and washing, 90 μl of substrate solution was added to each well, and the plates were incubated in the dark for 15–30 min at 37 °C. The reaction was stopped by adding 50 μl of stop solution to each well. The optical density (OD) of each well was measured at a wavelength of 450 nm within 5 min of adding the stop solution. A standard curve was generated from the OD values of the standard samples, and the concentrations of the proteins in the test samples were calculated accordingly. All plasma biomarker assays were performed in duplicate and averaged.

### Statistical analysis

Data analysis was performed using SPSS software, version 26.0. The Shapiro–Wilk test was applied to determine the normality of the continuous variables. Continuous data that followed a normal distribution across the three groups were analyzed using one-way analysis of variance (ANOVA) followed by Bonferroni post hoc test, and the results were reported as means ± standard deviations. For continuous data that did not follow a normal distribution, the Kruskal–Wallis rank sum test was utilized, with results presented as medians with the 25th and 75th percentiles [M (P25, P75)]. Categorical data were compared using the chi-square test.

The diagnostic performance of the cognitive assessment scales and serum biomarker concentrations was evaluated using receiver operating characteristic (ROC) curve analysis. This method calculated the area under the curve (AUC) and determined the sensitivity and specificity of each test. GraphPad Prism version 8.0 was used for the generation of graphical representations of the data.

To investigate the relationships between serum NfL levels, age, and cognitive assessment scale scores across different groups, Spearman's rank correlation analysis was conducted. A *P*-value of less than 0.05 was considered statistically significant, indicating that the observed differences were unlikely to have occurred by chance.

### Ethical approval

All participants had signed informed consent forms, and the study was approved by the Ethics Committee of the First Hospital of Hebei Medical University (Ethical Approval Number: 20190416).

## Results

### Demographic comparisons

The demographic analysis revealed no significant differences in gender, age, or educational level among the HC, MCI, and probable AD groups, ensuring comparability of the demographic data. The prevalence of hypertension and hyperlipidemia was also similar across the groups. However, significant differences emerged in the history of smoking, alcohol consumption, diabetes, and coronary heart disease among the participants (Table 1).

**Table 1:** Demographic comparisons among the three groups[(‾Χ ± S)/M(P25, P75)/n(%)].

	HC	MCI	Probable AD	*F/X2/Z*	*P*
	(n = 97)	(n = 44)	(n = 16)		
Gender					
Men	48.0 (49.5)	20.0 (45.5)	6.0 (37.5)	0.861	0.638
Women	49.0 (50.5)	24.0 (54.5)	10.0 (62.5)		
Age	68.7 ± 4.0	72.6 ± 8.0	70.6 ± 8.7	5.426	0.066
Education level					
Illiteracy	9.0 (9.3)	2.0 (4.5)	3.0 (21.4)	4.419	0.356
Primary school	16.0 (16.5)	5.0 (11.4)	1.0 (6.3)		
Junior high school and above	72.0 (74.2)	37.0 (84.7)	12.0 (75.0)		
Smoking history					
Never smoked	64.0 (66.0)	16.0 (36.4)	13.0 (75.0)	12.924	0.011*
Have not quit smoking	8.0 (8.2)	7.0 (15.9)	1.0 (6.3)		
Quit smoking	25.0 (25.8)	21.0 (47.7)	3.0 (18.8)		
Drinking history					
Yes	37.0 (42.0)	30 (45.5)	5.0 (12.5)	15.769	0.015*
Never	60.0 (61.9)	14.0 (31.8)	11.0 (68.8)		
Hypertension					
No	49.0 (50.5)	21.0 (47.7)	10.0 (62.5)	1.044	0.601
Yes	48.0 (49.5)	23.0 (52.3)	6.0 (37.5)		
Hyperlipemia					
No	67.0 (69.1)	27.0 (61.4)	13.0 (81.3)	2.237	0.341
Yes	30.0 (30.9)	17.0 (38.6)	3.0 (18.8)		
Diabetes					
No	69.0 (71.1)^a^	20.0 (45.5)^b^	11.0 (68.8)^ab^	8.829	0.012*
Yes	28.0 (28.9)^a^	24.0 (54.5)^b^	5.0 (31.3)^ab^		
Coronary heart disease					
No	77.0 (79.4)^a^	24.0 (54.5)^b^	14.0 (87.5)^ab^	11.375	0.004**
Yes	20.0 (20.6)^a^	20.0 (45.5)^b^	2.0 (12.5)^ab^		

Educational attainment: The corner scale ^abc^ indicates that there was a difference between the two groups, a and b represent differences between HC and MCI; *denote *P* < 0.05, ** denote *P* < 0.01.

Significant values are in [bold].

### Cognitive function comparisons

Cognitive function assessments among the HC, MCI, and probable AD groups revealed significant differences in performance on the MoCA, MMSE, BNT, and DST. Both the MCI and probable AD groups scored significantly lower than the HC group on all cognitive tests, indicating a decline in cognitive function associated with disease progression (Table 2).

**Table 2:** Comparison of cognitive function among the three groups.

	HC group	MCI group	Probably AD group	*F*	*P*
	(n = 97)	(n = 44)	(n = 16)		
MoCA	27.0 (25.5, 28.5)	23.0 (20.3, 24.0)	19.0 (14.0, 22.8)	57.46	< 0.001**
MMSE	29.0 (27.0, 30.0)	27.0 (25.0, 27.0)	22.0 (17.3, 23.8)	95.38	< 0.001**
BNT	26.0 (24.0, 28.0)	26.0 (24.0, 28.0)	22.5 (17.8, 24.8)	10.79	< 0.001**
DST	9.0 (7.50, 10.0)	8.5 (7.0, 10.0)	6.00 (5.0, 7.8)	10.80	< 0.001**

MoCA: Montreal Cognitive Assessment; MMSE: Mini-mental state examination; BNT: Boston naming test; DST: Digit span test; ** denote *P* < 0.01.

### Serum biomarkers comparison

Upon comparing serum biomarkers among the HC, MCI, and probable AD groups, no significant differences were found in levels of IL-1β, TNF-α, and p-tau217. However, a notable difference was observed in serum IL-6 concentrations (F = 8.752, *P* = 0.013), with the MCI group exhibiting the highest levels, followed by the probable AD group, and the lowest in the HC group. Serum NfL concentrations also differed significantly across the groups (F = 7.582, *P* = 0.023), with the MCI group showing higher levels than the HC group. The MCI group's NfL levels were higher than those of the probable AD group, although this difference did not reach statistical significance. Aβ40 concentrations varied significantly among the groups, with the MCI group having the highest levels. Serum Aβ42 concentrations were significantly different across the groups, with the highest levels in the HC group and a significant difference between the HC and MCI groups. The Aβ42/Aβ40 ratio did not show a statistical difference (Table 3).

**Table 3:** Comparison of blood indicators among the three groups.

	HC group^①^(n = 97)	MCI group^②^(n = 44)	Probable AD group^③^(n = 16)	*H*	*P*	Pairwise comparison
IL-1β (pg/mL, P25, P75)	0.7 (0.4, 1.3)	1.0 (0.4, 2.2)	0.8 (0.4, 1.2)	3.49	0.18	
IL-6 (pg/mL, P25, P75)	1.1 (0.8, 1.6)	1.5 (1.0, 2.3)	1.3 (1.0, 2.0)	8.75	0.013*	① < ③, ① < ②*, ③ < ②
TNF-α (pg/mL, P25, P75)	3.3 (2.1, 5.7)	2.6 (1.6, 4.3)	4.2 (1.8, 6.2)	2.39	0.30	
NfL (pg/mL, P25, P75)	1591.8 (1145.4, 2007.1)	1932.1 (1352.4, 2778.7)	1632.1 (1082.2, 2245.2)	7.58	0.023*	① < ③, ① < ②*, ③ < ②
Aβ40 (pg/mL, P25, P75)	32.2 (7.1,76.8)	157.9 (24.4, 242.7)	23.6 (7.2, 70.6)	17.00	*P* < 0.001**	③ < ①, ③ < ②*, ① < ②*
Aβ42 (ng/mL, P25, P75)	0.7 (0.4, 1.1)	0.2 (0.1, 0.9)	0.6 (0.4, 0.9)	7.31	0.026*	② < ①*, ② < ③, ③ < ①
p-tau217 (pg/mL, P25, P75)	685.3 (521.3, 871.9)	795.3 (576.2, 871.9)	624.9 (436.9, 782.6)	5.70	0.06	
Aβ42/Aβ40	28.5 (7.2, 81.3)	0.8 (0.6, 39.5)	46.0 (14.0, 90.6)	0.80	0.45	

IL-1β: Interleukin 1β; IL-6: Interleukin 6; TNFα: Tumor necrosis factor α; NfL: Neuroflament light chain; Aβ40: β amyloid 40; Aβ42: β amyloid 42; * denote *P* < 0.05, ** denote *P* < 0.01.

### Correlation analysis between cognitive and hematological indicators

Spearman correlation analysis explored the relationships between serum NfL levels, inflammatory factors, and cognitive assessment scale scores. Age showed a positive correlation with IL-1β and Aβ40, but a negative correlation with Aβ42 and MMSE scores. Serum NfL was positively correlated with IL-1β and IL-6, as well as Aβ40, but no significant correlation was found with cognitive assessment scores. TNF-α correlated with Aβ42, Aβ40, and p-tau217. Aβ40 showed a positive correlation with DST scores (correlation coefficient = 0.288). Significant positive correlations were observed among the cognitive measurement scales (Table 4).

**Table 4:**
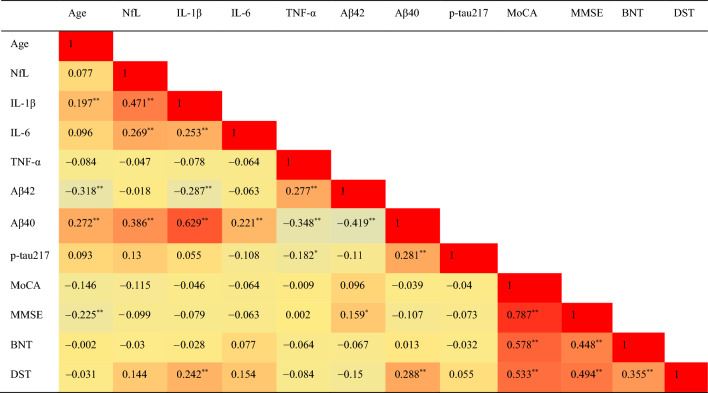
Correlation analysis of age, cognitive assessment and blood indicators in all populations.

IL-1β: Interleukin 1β; IL-6:Interleukin 6; TNFα:Tumor necrosis factor α; NfL: Neuroflament light chain; Aβ40: β amyloid 40; Aβ42: β amyloid 42; MoCA: Montreal Cognitive Assessment; MMSE: Mini-mental state examination; BNT: Boston naming test; DST: Digit span test; * denote *P* < 0.05, ** denote *P* < 0.01.

### Sensitivity and specificity analysis of indicators predicting MCI

**Figure 1:**
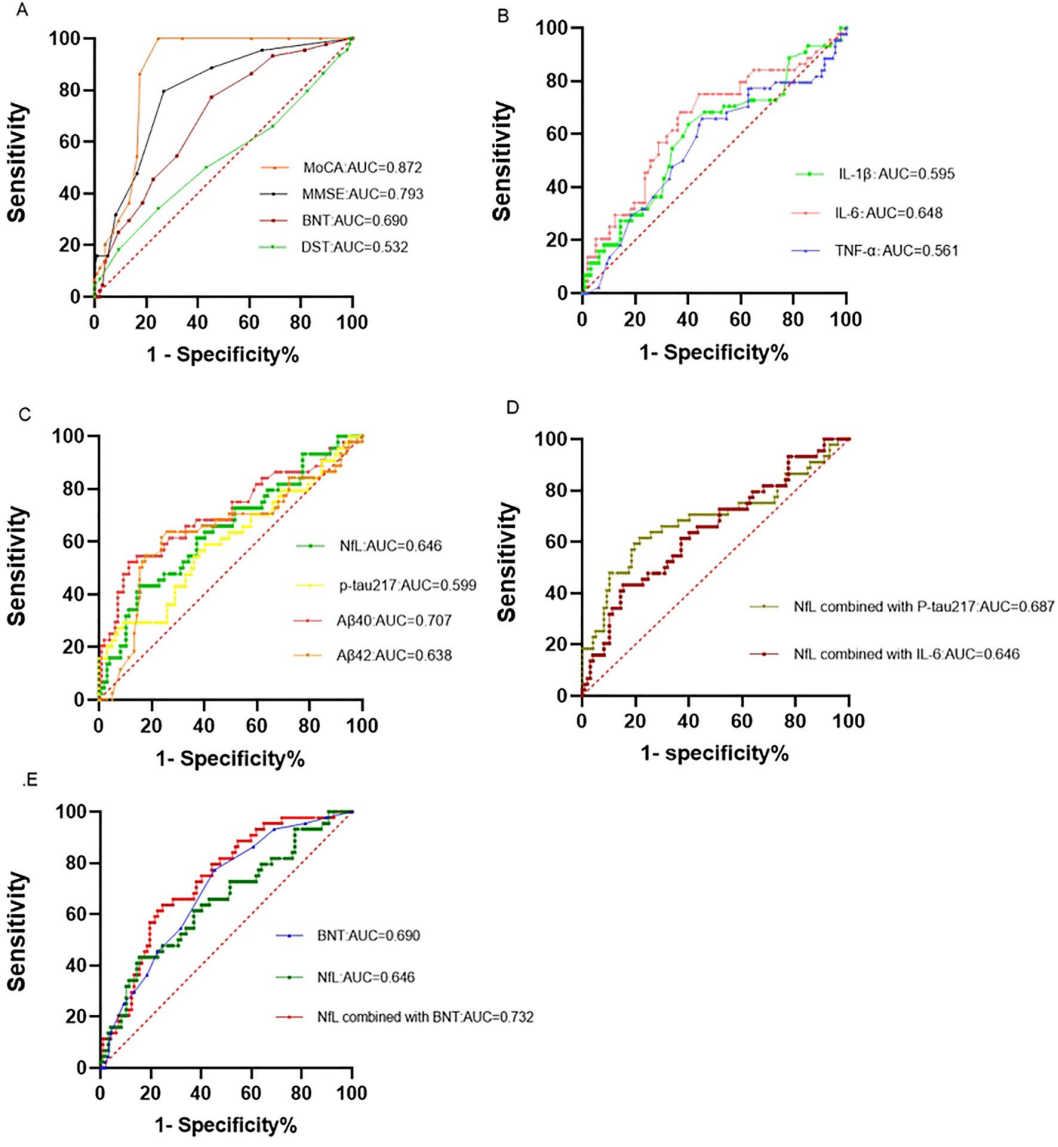
ROC analysis of different cognitive scales and blood indexes.Note: MoCA—Montreal Cognitive Assessment; MMSE—Mini-mental state examination; BNT—Boston naming test; DST—Digit span test; IL—Interleukin; TNFα—Tumor necrosis factor α; NfL—Neuroflament light chain; Aβ—β amyloid; p-tau—phosphorylated tau.

ROC curve analysis assessed the sensitivity and specificity of various blood and cognitive tests for predicting MCI. The BNT showed an AUC of 0.690, with a sensitivity of 0.773 and a cutoff value of 25.5. The DST had limited predictive ability, with an AUC of only 0.546(Fig. 1A). IL-6 demonstrated higher accuracy in predicting MCI compared to IL-1β and TNFα, with AUCs of 0.648, 0.595, and 0.562, respectively (Fig. 1B).

Serum NfL, Aβ42, Aβ40, and p-tau217 showed high specificity in predicting MCI, with Aβ40 outperforming the others (AUC = 0.707). The AUC for NfL was 0.646, and although p-tau217 had a lower AUC of 0.599, its specificity reached 0.907(Fig. 1C). Combining NfL with p-tau217 increased the predictive accuracy (AUC = 0.687), but combining NfL with IL-6 did not improve accuracy beyond that of NfL alone. The combination of NfL and BNT increased the accuracy of MCI prediction (AUC = 0.732) (Fig. 1D,E).

### Gender differences in predicting MCI

Analysis of gender differences in the predictive accuracy of blood indicators and cognitive tests for MCI revealed that IL-6 predicted the highest AUC value for MCI in men. The combination of NfL and BNT improved the accuracy of identifying MCI in men (AUC = 0.777). In contrast, TNF-α was a more accurate predictor for MCI in women than other inflammatory indicators, while IL-6 was not a significant predictor for MCI in women. However, The AUC value of NfL combined with BNT for identifying MCI was not higher than BNT alone for in women (Table 5).

**Table 5:** Gender differences in MCI sensitivity and specificity with different blood and cognitive measures.

	Men (n = 68)					Women (n = 73)				
	AUC	Cut off	Sensitivity	Specificity	*P*	AUC	Cut off	Sensitivity	Specificity	*P*
IL-1β	0.525	2.12	0.132	0.682	0.747	0.661	0.92	0.750	0.644	0.026
IL-6	0.719	1.33	0.800	0.667	0.005	0.596	0.58	0.957	0.875	0.185
TNF-α	0.564	1.50	0.912	0.86	0.408	0.677	3.26	0.792	0.592	0.023
NfL	0.663	2016.7	0.450	0.875	0.035	0.625	2156.7	0.458	0.826	0.085
BNT	0.692	27.5	0.900	0.417	0.013	0.700	23.5	0.667	0.653	0.006
NfL + IL-6	0.719	0.51	0.800	0.667	0.005	0.500	0.24	0.875	0.407	0.040
NfL + BNT	0.777	0.47	0.800	0.708	*P* < 0.001	0.700	0.31	0.667	0.653	0.006

IL-1β: Interleukin 1β; IL-6: Interleukin 6; TNFα: Tumor necrosis factor α; NfL: Neuroflament light chain; BNT: Boston naming test.

## Discussion

The NfL protein, a neuron-specific element of the neuronal cytoskeleton, has been identified as a biomarker for neuronal damage, with elevated levels in CSF and blood linked to various neurological conditions, including multiple sclerosis, frontotemporal dementia, and Guillain–Barre syndrome^26,27^. Additionally, research has shown that higher CSF NfL concentrations correlate with compromised white matter integrity, as assessed by diffusion tensor imaging, suggesting that white matter damage is a primary contributor to memory decline in MCI^28^. Recent hypotheses propose that AD may be a chronic autoimmune disease, with cognitive decline closely associated with inflammatory processes^17^. Our study sought to explore the relationship between serum NfL, inflammatory factors, and cognitive function.

We observed that serum NfL levels were elevated in both the MCI and AD groups compared to the HC group, with the highest concentrations found in the MCI group. This aligns with previous findings^29^ and suggests that serum NfL levels begin to rise even before significant cognitive decline or impairment in daily living activities becomes apparent, offering potential implications for drug development and early intervention strategies. Longitudinal studies have indicated that higher baseline NfL levels are predictive of more rapid cognitive deterioration in individuals with MCI^30^. Furthermore, increases in plasma NfL levels have been associated with structural brain changes, including alterations in gray matter, white matter, and the cingulate gyrus, as well as with the volume of the lateral ventricles, hippocampus, and cortical thickness in AD patients^31,32^. The interaction between inflammatory processes and plasma NfL may also influence cognitive integrity through the disruption of core subsystems involved in AD progression and the frontoparietal network^33^.

In our study, serum IL-6 levels differed significantly across the groups, and IL-1β levels correlated with DST scores, suggesting that monitoring these cytokines could aid in the early detection of MCI in at-risk populations. The serum Aβ40 levels were higher in the MCI group and lower in the AD group, while serum Aβ42 levels were lowest in the MCI group and higher in the AD group compared to the MCI group. These findings contrast with some previous studies where Aβ40 and Aβ42 levels were found to be lower in MCI than in HC groups^34^. Other research indicates that plasma Aβ levels may decrease significantly during the AD stage, potentially due to changes in the blood–brain barrier permeability, lymphatic function, or activation of vascular components or microglia, leading to reduced clearance of Aβ from the CSF to the periphery^35,36^. Animal studies support this, suggesting that blood Aβ levels drop as Aβ begins to deposit in the brain^37^. The discrepancy in Aβ40 and Aβ42 levels observed in our study may reflect complex and as yet not fully understood mechanisms of Aβ production and clearance.

While previous research has indicated an association between age and NfL concentration^38^, our study did not observe this relationship. This discrepancy may be attributed to the different methodologies employed for quantifying blood NfL, such as ELISA, electrochemiluminescence immunoassay, and Single Molecule Array (Simoa), with Simoa being the most sensitive^39^. Due to its low cost, convenient operation, and the ability of key reagent antibodies to specifically bind to its target, ELISA has a low off target rate, which can produce high sensitivity and specificity results. Moreover, antibodies validated by ELISA can be replicated on a large scale. Previous studies have also used ELISA to detect NfL levels, a study utilizing ELISA also reported no correlation between age and NfL levels^40^. This suggests that the choice of detection method could significantly impact the observed correlations between NfL and age.

Our investigation into the correlation between NfL and inflammatory cytokines revealed a positive association between NfL and IL-1β and IL-6. This aligns with animal studies that have documented an increase in various inflammatory factors, including IL-2, IL-4, IL-6, and TNF-α, in conjunction with elevated CSF NfL in the context of chronic neuroinflammation and immune response^41^. These findings lend credence to the hypothesis that AD may be viewed as an innate autoimmune disease^17^. The association between NfL and Aβ40 suggests that axonal damage may play a significant role in the AD disease process, alongside the well-established roles of Aβ and tau pathology.

The relationship between inflammation and cognitive decline is widely acknowledged, yet the precise mechanisms and pathways of brain inflammation remain elusive. In the early stages of AD, the brain's neuroprotective mechanisms, such as amyloid clearance and antioxidant defenses, are believed to be effective. However, as AD progresses, stress-related upregulation of immune system mediators leads to an overproduction of pro-inflammatory molecules, resulting in brain inflammation. The accumulation of Aβ and the formation of neurofibrillary tangles in the AD brain can activate the immune system and trigger inflammatory stress. This sustained inflammatory response can create a positive feedback loop, culminating in irreversible neuronal damage^42^. Thus, we hypothesized that combining NfL with inflammatory markers might enhance the accuracy of MCI detection. However, our study did not support this hypothesis, which may be due to the limited sample size. Nonetheless, combining NfL with the BNT or p-tau217 improved diagnostic accuracy, highlighting the multifaceted nature of AD pathology and the limitations of relying on a single biomarker.

Autopsy studies have demonstrated a strong correlation between plasma p-tau217 levels and the extent of neurofibrillary tangles in AD patients, with p-tau217 showing high diagnostic performance in differentiating AD from other neurodegenerative diseases^43^. Although NfL is a marker of axonal injury and lacks specificity, as axonal damage can result from various neurological conditions, it still holds value in the early identification of MCI. Our findings suggest that the combination of NfL with p-tau217 can enhance the diagnostic accuracy for MCI, offering a potential avenue for future diagnostic approaches in AD.

Our study contributes to the understanding of AD by demonstrating correlations between NfL and other AD markers. Although most correlations were not statistically significant, this is consistent with previous studies^32^, suggesting that AD pathology is driven by diverse pathological conditions, such as Aβ pathology, tau pathology, and axonal degeneration, each eliciting different biomarker responses. Overall, the correlations between these biomarkers were weak, indicating the complexity of AD’s pathophysiological mechanisms.

Gender-based analysis in our study revealed that the diagnostic accuracy of certain biomarkers for MCI is inconsistent between males and females, which could be attributed to the small sample size. However, this observation may also reflect inherent gender differences in inflammatory responses. Previous research has indicated that sex may influence the regulation of pro-inflammatory and anti-inflammatory biomarkers^44^, and sex differences in inflammation have been reported^45^. While some studies suggest sex disparities in NfL levels^46,47^, the evidence is not yet conclusive, and further research with larger sample sizes is necessary to elucidate these potential differences.

The strength of this study lies in its integrative approach, combining serum NfL with inflammatory markers and other pathological indicators of AD, thereby shedding light on their interrelationships within the complex AD pathology. The inclusion of an elderly Eastern population provides valuable insights that are pertinent to our regional context and diversifies the demographic representation in AD research. Nevertheless, our study has limitations that warrant consideration. The small sample size and the regional confinement to Hebei Province may limit the generalizability of our findings. Additionally, the cross-sectional design precludes the ability to observe dynamic changes over time in the relationship between serum NfL, inflammatory markers, and cognitive function. The absence of neuroimaging data also means that we could not explore the association between serum NfL levels and brain structural changes.

## Conclusion

This study underscores the potential of serum NfL as a biomarker for the early detection of MCI and AD, with elevated levels observed in affected individuals compared to healthy controls. The positive correlations between serum NfL and inflammatory cytokines IL-1β, IL-6, as well as Aβ40, highlight the multifaceted nature of AD pathology. While the combination of serum NfL with p-tau217 and the BNT enhances the predictive accuracy for MCI, the addition of inflammatory markers does not yield further improvement. These findings suggest that serum NfL, particularly when combined with other specific biomarkers, could serve as a valuable tool in the early identification of AD, thereby informing potential therapeutic strategies and interventions.

## Data Availability

The datasets generated during and/or analysed during the current study are available from the corresponding author on reasonable request.
